# Retraction: *Tropheryma whipplei*: A common bacterium in rural Senegal

**DOI:** 10.1371/journal.pntd.0013229

**Published:** 2025-06-26

**Authors:** The PLOS Neglected Tropical Diseases Editors

The *PLOS Neglected Tropical Diseases* Editors retract this article [1,2] due to concerns about compliance with the PLOS Human Subjects Research policy.

The Materials and Methods section in [1] reports sampling of saliva and stool specimens from the population of Dielmo and Ndiop, Senegal, in April and July-October 2009. In addition, the study reports the inclusion of serum samples collected in May, June, and July of 2008 and May of 2009. The ethics approval reported in the article refers to agreement #09–022 issued by the Ethic Committee of the Institute Fédératif de Recherche 48 (IFR48), Marseilles, France. The article does not report prospective, local ethics approval from an institution or agency in Senegal. Furthermore, PLOS noted that the ethics approval number #09–022 was also reported in at least 247 other studies despite apparent differences in the aims and objectives, study locations, study populations, age ranges, methodologies, types of samples collected, and types of consent described in these studies. S1 File contains a summary of articles citing ethics approval number #09–022 of which PLOS is aware.

Co-author JFT responded stating that 90% the inhabitants of Dielmo and Ndiop have been collaborating with the research teams in order to improve their state of health since 1993 and continued to do so in 2024. However, the author did not provide local ethics approval documentation for editorial review.

A representative of the Aix-Marseille Université Ethics Committee stated that the institute disagrees with the retraction decision, and that the study complied with applicable legislation and ethical standards. They stated that the study reports a retrospective analysis of previously collected samples, and that the ethics opinion #09–022 quoted in the article allows the retrospective application of an analytical method to samples previously acquired. The representative also commented that the authors collaborated on a long-term project that started in 1993, and provided multiple favorable opinions from local ethics committees for other studies caried out in Senegal, but they indicated that the institute was unable to locate the original ethics approval document due to the age of the study.

PLOS received a copy of the ethics approval document #09–022 issued by the IFR48 separately from the inquiries on this case, and concluded that the documents and the institute’s response did not resolve the concerns. Specifically,

- The document #09–022 was issued on December 9, 2009, after the sample collection dates reported in [1]. The *PLOS Neglected Tropical Diseases* policies for Human Subjects Research states that studies involving human participants must obtain prior ethics approval.
- The document #09–022 provides approval for the use of stool samples only, and lists the “Bacteriological laboratory of the Timone Hospital, Marseille, France” as the origin of the samples.
- The additional ethics documentation provided by the institute demonstrated approval for studies that did not match the topic, location, sample collection period, or type of samples reported in this article [1]. PLOS has not received any prospective local ethics approval documentation supporting the collection of stool, saliva, and serum samples for the study reported in [1], nor has PLOS received any evidence to confirm local ethics approval for the secondary analysis of previously collected stool, saliva, and serum samples as part of a separate study.

In addition, PLOS identified potential competing interests between the approving ethics committee that issued the document #09–022 and one or more of the article’s authors.

CS, JFT and DR did not agree with the retraction. AKK, HB, AT, PR, and FF either did not respond directly or could not be reached.

## Supporting information

S1 File Overview of 248 articles referencing ethics approval number #09–022. (XLSX)
